# The Life of Jorjani: One of the Persian Pioneers of Medical Encyclopedia Compiling: On the Occasion of His 1000th Birthday Anniversary (434, A.H. - 1434, A.H.)

**DOI:** 10.5812/ircmj.8080

**Published:** 2013-09-05

**Authors:** Fariborz Moattar, Mohammad Reza Shams Ardekani, Alireza Ghannadi

**Affiliations:** 1 Department of Pharmacognosy, Faculty of Pharmacy and Pharmaceutical Sciences Research Center, Isfahan University of Medical Sciences, Isfahan, IR Iran; 2 School of Traditional Iranian Medicine, Tehran University of Medical Sciences, Tehran, IR Iran

**Keywords:** Jorjani, Islamic Traditional Medicine, Iranian Traditional Medicine, Zakhireh Khwarazmshahi

## Abstract

**Background:**

Seyyed Esmaeil Jorjani is one of the most prominent pioneers and scientists of Islamic and Iranian traditional medicine in the 11^th^ and 12^th^ centuries (4^th^ and 5^th^ centuries A.H.). The number of his books is not certainly clear, but it is signified that he has written a couple of great books concerning medicine for Persians and too many treatises in such fields as philosophy, theology, medical ethics, human anatomy, chemistry, pharmacy and other sciences. His most famous and important book is “Zakhireh Khwarazmshahi” or “The Treasure of King Khwarazm”, which is really a complete and valuable medical encyclopedia in Persian language.

**Materials and Methods:**

In the present study, authors have attempted to state the life and time of Jorjani and his views by studying the history, old medical sources, and other provided recent medical literature in these fields.

**Results:**

The biography of Hakim Jorjani has been well reviewed and described precisely.

**Conclusions:**

Based on our findings, it is clear that Jorjani described and explained the symptoms, signs and treatments of several diseases, introduced the activities of various medicinal plants, and compound formulations. He achieved all of this during his continuous visits to prestigious medical centers and famous people of his time.

## 1. Introduction

There is a limited knowledge about the history of science and medicine in old Iran, unlike its deep 10000 years of history and historical evidences. Persian civilization is one of the oldest living civilizations (1-3). Persian roles as pioneers who have attempted to transfer their fundamental theories, believes and experiments to next generations, so that the generation of medical and scientific knowledge remains known (2, 4). Among Persian talented scientists, Seyyed Esmaeil Jorjani is one of the most famous physicians of the Islamic and Iranian traditional medicine. He is among the scientists whom despite great fame, little is known about their lives nowadays. (4-6). Jorjani’s name is a style, which shines in the Islamic and Iranian history of medicine. At first, in order to be more familiar with this outstanding physician, it might be better to recall some of the titles and phrases attributed to him by contemporary scholars or scientists who wrote in his tribute and honor: “The crown of cleanliness"; “The star of Islam,”; “The spirit healer”; “The supreme of the wise”; “The resuscitator of medicine and other sciences,”; “The editor of rules and The cost of medicine” (6, 7).

All of these gimmicks represent the fame pinnacle of this physician from centuries ago up to the present time.

## 2. The life of Jorjani

Seyyed Zeynoddin (Sharafoddin) Abu Ebrahim Esmaeil Ibn Hassan Ibn Mohammad Ibn Ahmad Al-Hosseini Al-Jorjani (Gorgani) was born in 434, A.H. (1042, A.D.) in Jorjan (the northeast of Iran near the shores of the Caspian Sea, the largest lake in the world). A.H. stands for “After Hijrat”, referring to the Muslim calendar, and A.D. stands for “Anno Domini” that its Latin meaning is “In the Year of Our Lord” and referring to the Gregorian calendar system. It is said that Jorjani was from the Sadat Hosseini of Isfahan in such a way that he was also named Sepahani-e Jorjani (6). However, there is no exact information about his family, ancestors and when and why they have emigrated from Isfahan to Jorjan. Not much information is available concerning his childhood and education; It can be assumed that he has started his early education in Jorjan. On the other hand, it is unclear to say that his medical education has started in Jorjan (8, 9). Jorjan is the city where Ibn Sina (Avicenna) lived, taught and even wrote his first book the so called “Canon of Medicine.” There were also other scientists in Jorjan who had been engaged to teach several sciences (8). During that time, Jorjan had a good scientific stature as a city with advanced parties and scholars who were talking about science everywhere.

Seyyed Esmaeil Jorjani in addition to being proficient in medical and pharmaceutical sciences, like most of his great predecessors and forerunners such as Abubakr Mohammad Ibn Zakaryya Razi (Rhazes) and Ibn Sina, he was also a capable practiced in theological, philosophical and ethical sciences. Thus, it can be said that Jorjani probably has begun to learn medicine and Hekmat from his tutors in Jorjan (1, 3, 7).

Later, he travelled from Jorjan to Neishaboor and then went to different parts of Fars, Isfahan, Ghom, Khorassan and Khouzestan provinces. He benefited from the physicians over there too. At that time, Neishaboor was the great center of science and there were great colleges and sanatoriums (8). Cities such as Ray (with the presence record of Razi in it) and Isfahan (with presence record of 14 years of Ibn Sina and Ibn Mandouyeh) were among other cities, which Jorjani had visited in his life, and both of them had famous schools (6). His Isfahanian background, family history and anticipation of his relative visiting may have been the reasons of traveling to Isfahan and famous hospitals in Shiraz which from Abu Maher Musa Ibn Sayyar times were proofs for Shiraz visiting. Jorjani travelled to Khouzestan province several times as well, although the old international University of Jondishapour was abeyant for a while, in spite famous and excellent physicians were there. The ancient city of Jondishapour provided a unique and peaceful meeting point for the Persian, Greek and Indian philosophical and medical traditions (7). Jorjani also visited Ghom. At his time, Ghom has been known as the city of Shiite Muslims. There were several tutors in Ghom who taught various sciences (6, 7).

This great and eminent scientist completed his studies under the guidance of “The Second Hippocrates”, or Abu Al-Ghasem Abdul Al-Rahman Ibn Ali Ibn Abi Sadegh Al-Neishaboori, who was one of the academic and serious scholars of Ibn Sina. Ibn Abi Sadegh played an incomparable role in his education, where according to Ghotbeddin Lahiji and Ibn Abu Assibia statements, he had a brilliant intellect and was capable in philosophy, morality, ethics and medicine. Ibn Abi Sadegh has been considered as one of the greatest hakims concerning human anatomy sciences too (1, 6, 10). In this way his superior knowledge and studiousness particularly in the field of anatomy is manifested and the result of these precious information and knowledge is evident in his later writings (9-11). He made a comprehensive study of Galen’s books as well. After graduation, Jorjani continued his further studies under the guidance of “Second Hippocrates” during which he met with many scientists and Ibn Sina's students. In the year 504, A.H. (1110, A.D.) he returned back to the northern Persian province of Khwarazm (6-8). He explained about traveling to Khwarazm and undertaking of writing his great book, “Zakhireh Khwarazmshahi”. The introduction written for this book is as follows: “Since it was destined by the most high that the author of this book should ever pray for Sultan Khwarazmshah on account of the bounties that he has received from him, a ruler most educated and just and stepping stone to success, the protector of Islam and Muslims, the destroyer of unbelievers and rafezies, the safeguard of Faith and the honour of his subjects, the greatest of all sultans, Arsalan-e Takin Yamin Almuluk Abulfath Mohammad Ibn Yamineddin and Moein Amir Al-Moeminin, may God continue his crown and kingdom and make firm the basics of his rule. I travelled finally to Khwarazm and enter into his auspicious service, and this was in the year 504, A.H. I saw the healthful climate of Khwarazm and observed the manner and policies and justice of this great sultan. Regarding to his nice characterization, I have afforded and decided to live here and enjoy life under the kind and just rule, and always be grateful to him and to serve sultan sincerely in a spirit of faithfulness and to publish of my scientific projects upon the composition of which my whole lifetime was acquired knowledge. It was in accordance with this design that the book materials were gathered and named the “Zakhireh Khwarazmshahi” that the memory of this kind sultan may live forever in people's minds and his approval ever increase around the world.” The book was composed in such a specific and detailed Persian. He wrote it during a specific socio-political situation of Khwarazm (7, 8, 12).

According to Yaghut Al-Hamavi Al-Rumi’s statement in this regard, who was an Islamic biographer and geographer, Jorjani acquired the Hadith sciences from Abu Al-Ghasem Ghoshairi, the famous scientist of Neishaboor. Most probably, Jorjani prior to his trip to Khwarazm, he remained in Marv for a long time where he composed the book entitled “Zakhireh Khwarazmshahi” and most of his other writings and compilations while he was at the service of Ghotbeddin Mohammad. After sultan’s death, Jorjani spent the last years of his life in kindness of Alaoddowleh atsœz, the sultan’s son, who greatly admired Jorjani (8, 9, 13). Khorassan at that period was considered as one of the most important centers of Islamic and Iranian culture and civilization and was divided into four large parts: Marv, Harat, Neishaboor and Balkh (1, 6). The presence of Jorjani in Neishaboor was in the beginning of his adolescence and it seems that his stay in Marv and Balkh was probably in his middle ages. It is also possible that he involved in practicing and teaching in these two cities as well. Beihaghi who was a famous hadith expert, mentioned that he had visited Jorjani in the year 531, A.D. at the height of knowledge in Sarakhs, which is located between Mashhad and Marv. Sarakhs was a famous stopping point along the Silk Road during Jorjani’s time, had many bibliothecs and a famous academy of architects (1, 3, 6, 8). Jorjani spent the last remaining years of his life in Marv, teaching medicine and treating patients. Jorjani died at the age of 94 (in the year 531, A.H. 1136, A.D.) in Marv and during Khwarazmshahi dynasty (9, 14, 15).

Jorjani’s outstanding contribution to the improvement of knowledge in medicine, pharmacy and medical laboratory sciences is honoured in Tehran University of Medical Sciences, Iran by prizing sponsorship and research grants to the university academics with “Hakim Jorjani” Prize and medals in an annually scientific festival (16). In Iran, Jorjani's birth date (Iranain Month: Farvardin 30th equal to April 18th) is celebrated as the national medical laboratory medicine and sciences’ day (14).

## 3. Books and Compilations of Jorjani

The exact number of the books and writings of Hakim Jorjani has not been mentioned in the bibliography of the history of medicine. During Jorjani’s time, medicine and pharmacy in Iran switched to the Persian language and encouraged the learning and teaching of this science. He compiled a couple of books concerning medicine in Persian language and an innumerable amount of others in fields such as philosophy, medical ethics, theology, human anatomy, chemistry, pharmacology, pharmacy and other natural sciences (7, 8, 12, 17, 18). During his traveling to the different parts of Iran Jorjani described and explained the humoral theories, accurate diagnosis, symptoms, signs and treatments of various ailments, introduced the pharmacological activities of various medicinal plants, nutraceuticals and other natural products and clarified the six essential factors for life.. In clarification of these six factors, Jorjani also explained the importance of climate and influence of the environment on health in details. He may have also been one of the first hakims and physicians who gave a complete and full description and explanation of medicine disciplines and conditions like cancer and tumors, surgery and neurosurgery, dentistry, cosmetics, ulceration, urology, gynaecology, endocrinology, ophthalmology, midwifery, neonatal and childhood cares, geriatrics, sport and preventive medicines (12-15, 19-24). Jorjani’s innovations in several branches of medicine were novel and unique. Several of his theories and comments were not repeated until 18th and 19th centuries by world physicians. Some of his views are in line with current scientific views (6, 21, 24, 25).

His two important books are:

1. Zakhireh Khwarazmshahi: “Zakhireh Khwarazmshahi” is Jorjani’s most important book that has been written in Persian language with nearly 750000 words. It might be considered as the oldest and long-established Persian medical encyclopedia. He dedicated this book to Ghotbeddin Khwarazmshah. At the age of 70 with an ample and broad knowledge of medicine and other natural sciences, Jorjani wrote this masterpiece from his own experiences, thoughts and the writings of several previous physicians. It has been standing along with Ibn Sina’s “Canon of Medicine”. Arrangement of “Zakhireh Khwarazmshahi” volumes is very similar to the “Canon of Medicine”. His treatise was the most popular medical atlas in the Safavid dynasty of the 17th century, more than 600 years after its compiling (1, 3, 9, 13, 14, 16, 25, 26).

In fact, this distinguished treasure is a supreme encyclopaedia of medical sciences, which consists of eleven books. He compiled it in nine treatises but as referred at the end of the book he changed his judgment, in the last volume entitled “Gharabazin”, he added two more volumes dealing with the anatomy of the human body, especially neuroanatomy, and helpful introductions to the various parts of animals’ organs (6, 8).

Several archives of “Zakhireh Khwarazmshahi” exist in the world, Iran and couple of volumes of this book have been published in Iran by Iranian Academy of Medical Sciences and other publishers. It was translated to various languages like Turkish, Arabic, Urdu and Hebrew (12, 14, 26).

2- Khoffi-e Alaei: This book is a brief and compact form of the “Zakhireh Khwarazmshahi” that included a short review of the science of medicine. Jorjani wrote this medical manual at the request of Amir Isfahar Arsalan, the grantee prince of Abu Al-Muzaffar atsœz Khwarazamshahi. In the first chapter of the book, Jorjani composed “I prepared “Khoffi-e Alaei” as a summary and pocket book form “Zakhireh Khwarazmshahi” which can be easily read and skim at almost all times even travelling, wars and battles” (8, 18).

The exact date of the composition of “Khoffi-e Alaei” has not been recorded. Jorjani mentioned it clear that it was written after the year 504, A.H. (1110, A.D.) that is after the precise composition of “Zakhireh Khwarazmshahi” (8, 18). One of the versions of this book that exists in Typo Sultan's Library was published in 507, A.H. (1113, A.D.). There are also some documents that indicated it was published after the year 522, A.H. (6, 7). This book has been edited and analyzed by several commentaries by Professor Dr. Mahmoud Najmabadi and Professor Dr. Ali Akbar Velayati 14 years ago, and was published by Ettelaat Publications in Tehran (18).

Conclusions: Jorjani was a great hakim, scientist, physician and pharmacist in Iran whose life and works were introduced by several authors. He is one of the pioneers of Islamic and Iranian Traditional Medicine. His contribution to the different fields of sciences gives back his great personality in the history of science in Islam and Iran. He gave a shape to the different systems of medicine and redefined several concepts based on his clinical experiences. Although a millennium has been passed from his birth date (434, A.H. - 1434, A.H.), his great role in the revival of Islamic and Iranian traditional medicine is incontestable yet. He belongs to all people of the earth, “Worldwide Jorjani.”
